# An evaluation of fish and invertebrate mercury concentrations in the Caribbean Region

**DOI:** 10.1007/s10646-024-02754-y

**Published:** 2024-06-05

**Authors:** Linroy D. Christian, Mark E. H. Burton, Azad Mohammed, Wendy Nelson, Tahlia Ali Shah, Laël Bertide-Josiah, Helen G. Yurek, David C. Evers

**Affiliations:** 1 Ministry of Foreign Affairs, Agriculture, Trade and Barbuda Affairs, St. John’s, Antigua and Barbuda; 2 Biodiversity Research Institute, Portland, ME USA; 3 The University of The West Indies, St. Augustine, Trinidad and Tobago; 4 Institute of Marine Affairs, Chaguaramas, Trinidad and Tobago

**Keywords:** Mercury, Caribbean, Contaminants, Biomonitoring, Fish, Invertebrates

## Abstract

Mercury is a ubiquitous pollutant of global concern but the threat of exposure is not homogenously distributed at local, regional, or global scales. The primary route of human exposure to mercury is through consumption of aquatic foods, which are culturally and economically important in the wider Caribbean Region, especially for Small Island Developing States (SIDS). We compiled more than 1600 samples of 108 unique species of fish and aquatic invertebrates collected between 2005 and 2023 from eleven countries or territories in the wider Caribbean Region. There was wide variability in total mercury concentrations with 55% of samples below the 0.23 µg/g wet weight (ww) guideline from the U.S. FDA/EPA (2022) for 2 or 3 weekly servings and 26% exceeding the 0.46 µg/g ww guideline consistent with adverse effects on human health from continual consumption, particularly for sensitive populations. Significant relationships were found between total mercury concentrations and taxonomic family, sampling country, fish length, and trophic level. The data analyzed here support the need for further sampling with concrete geospatial data to better understand patterns and mechanisms in mercury concentrations and allow for more informed decision making on the consumption of fish and invertebrates from the wider Caribbean Region as well as supporting efforts to evaluate the effectiveness of national, regional, and international mercury policies.

## Introduction

Mercury (Hg) is a ubiquitous, persistent pollutant of international concern (WHO 2020). It enters the environment through geogenic processes (e.g., volcanic activity, rock weathering), however, direct and indirect releases from anthropogenic activities far exceed natural releases (Driscoll et al. 2013; Streets et al. 2017; Obrist et al. 2018; Outridge et al. 2018; UNEP 2019). Mercury emitted into the atmosphere is transported at a broad spatial scale with anthropogenic activities estimated to have increased atmospheric mercury concentrations by 300–500% in the past century (Driscoll et al. 2013; Streets et al. 2017; Outridge et al. 2018; UNEP 2019). The Central America and Caribbean Region emits an estimated 45.8 tonnes of mercury per year (range: 37.2–61.4); primarily from the industrial sector (19.1 tonnes) and artisanal and small-scale gold mining (ASGM; 14.3 tonnes), which account for a combined 73% of total regional emissions (UNEP 2019). Additionally, mercury is released into water and land directly, through point sources, and indirectly, through deposition and remobilization of legacy mercury through human disturbance (e.g., deforestation) (Kocman et al. 2013, 2017; Diringer et al. 2020; UNEP 2019).

Once released or mobilized, the fate of mercury is complex and is persistently available as it cycles through ecosystems and species (Amos et al. 2013; Gustin et al. 2016; Eagles-Smith et al. 2018; Obrist et al. 2018). Inorganic mercury can be converted to methylmercury (MeHg), a neurotoxic and bioavailable form, through complex microbial processes (Gilmour et al. 2013; Hsu-Kim et al. 2013; Podar et al. 2015; Obrist et al. 2018). Methylmercury bioaccumulates in individuals and biomagnifies through food webs (Weiner et al. 2003; Wu et al. 2019). It impairs physiological and neurological function, particularly in fetuses and young children (Karagas et al. 2012; Basu et al. 2018; Evers 2018). In biota, elevated mercury concentrations increase the risk of reduced reproductive success, cause behavioral, physiological, and biochemical impairment, adversely impact growth and body mass, and can result in mortality (Webber and Haines 2003; Depew et al. 2012; Scheuhammer et al. 2012; Ackerman et al. 2016; Carvan et al. 2017; Evers 2018).

Exposure can occur through direct contact (e.g., use of elemental mercury in ASGM activities, application of mercury containing skin lightening products, occupational exposure) or indirectly (e.g., diet). Human exposure to methylmercury occurs predominantly through diet, primarily consumption of contaminated seafood (Sunderland 2007; Rice et al. 2014). Around 75–95% of total mercury (THg) in fish muscle is in the form of MeHg, although the percentage may be lower in smaller individuals (Grieb et al. 1990; Bloom 1992; Lescord et al. 2018). As a result, THg is widely used as a proxy for MeHg, and allows for comparison with established thresholds for human and biotic health (Evers 2018; Basu et al. 2018). However, fish provide vital nutritional benefits, with more than 3.3 billion people getting at least 20% of their animal protein intake from fish, and are particularly important in certain areas, including many Small Island Developing States (SIDS; FAO 2020).

Seafood consumption has changed significantly over the past four decades. Global fish consumption has increased from 9.6 kg per capita in the 1960s to 15 kg per capita in 2011 and exceeded 20 kg per capita in 2016 (FAO 2022). According to FAO (2022), while the average consumption of aquatic food in the Latin America and Caribbean Region was estimated to be 9.9 kg per capita per year in 2019, actual statistics may be higher, especially for SIDS. In the Caribbean Region, estimates of average fish consumption per person per year ranged from 2 kg in Belize to as high as 59 kg in Antigua and Barbuda, well above the global average, based on assumptions of consumption of available recorded fish (CRFM 2021).

For many Caribbean countries, fisheries are important culturally, as an essential, affordable, and accessible source of protein, and as an income source (Stephen and Murray 2008; CRFM 2021; FAO 2022; Maldonado et al. 2022). Research on fish consumption in the Caribbean Region and the associated mercury uptake has been ongoing to better inform consumption recommendations. An assessment of pregnant women in Jamaica and Trinidad and Tobago reported mercury concentrations varied based on the type of commonly consumed fish. Participants in the study living in countries mainly consuming pelagic fish had higher placental mercury concentrations than those primarily consuming reef fish (Ricketts et al. 2016). Preliminary regional- and national-scale fish consumption advisory guidelines for commonly consumed local fish have also been developed for several countries to help inform the public of species-specific tradeoffs between THg concentrations and health benefits (BCRC-Caribbean 2021). Although reliable data on small scale fisheries are often unavailable in developing countries, the wider Caribbean Region small-scale fisheries support a mean income considerably higher than the mean national income (Teh et al. 2020). The large-scale fisheries sector is also commercially valuable with total estimated aquatic catches in 2019 of 1.4 million tonnes in the Western Central Atlantic, including the Caribbean Sea (FAO 2022). It is estimated commercial marine fisheries in Caribbean Regional Fisheries Mechanism (CRFM) member states directly employed more than 118,000 people in 2019, indirectly accounting for more than 5% of the workforce and was worth 435 million USD (FAO 2010; CRFM 2021).

Several countries within the region are Party to the Minamata Convention on Mercury, which came into effect in 2017 and aims to protect human health and the environment from the anthropogenic effects of mercury at a global scale (UNEP 2013). Additionally, the regional Cartagena Convention (1983) adopted the “Protocol Concerning Pollution from Land-Based Sources and Activities” aiming to protect the marine environment of the wider Caribbean Region from pollution, including land-based sources. As part of the obligations of the Minamata Convention, Parties in the region have begun the process of implementing policies and drafting regulatory reforms to target the reduction of mercury releases. Recommendations of the Minamata Convention also include monitoring biotic media and vulnerable populations in order to evaluate the effectiveness of the required controls on mercury source emissions and releases and if subsequent reductions in emissions and releases translate to lower mercury concentrations in humans and biota. As a result, and in response to fish harvest and consumption patterns in the Caribbean, a Caribbean Region Mercury Monitoring Network (CRMMN) was formed in 2022. The CRMMN was constituted through agreement between national institutions in the wider Caribbean Region (Antigua and Barbuda, Belize, Guyana, St. Kitts and Nevis, St. Lucia, Suriname, and Trinidad and Tobago). The network seeks to collect mercury data to assess the risk to human and environmental health and will help define baseline mercury concentrations in the wider Caribbean Region in multiple matrices.

CRMMN biomonitoring is based on the Minamata Convention recommendation to use biosentinels in the evaluation of the effectiveness of measures taken to fulfill obligations of the convention. Four target fish species were selected to inform our understanding of different patterns and processes in the mercury cycle. Fish are commonly recognized as effective biosentinels of mercury exposure (e.g., Eagles-Smith et al. 2016) and the target species are commonly consumed to various degrees and are globally distributed for comparison to other regional networks. Additionally, differences in human mercury concentrations based on fish consumption patterns reported in Ricketts et al. (2016) informed the decision to have both reef and pelagic species represented. Yellowfin tuna are pelagic-oceanic predators (trophic Level=4.41). Mahi mahi are pelagic-neritic predators (trophic Level=4.21) that have also been identified by the FAO as a focal species to target for sustainable development of a fishery in the Dominican Republic (Beltrán Turriago et al. 2023). These species are integrators over larger regions as pelagic, migratory species. On the other hand, red snappers (trophic Level=3.75) and barracudas (trophic Level=4.27) are both reef-associated predators that can give a more localized spatial snapshot.

The collection of the baseline data in this region is especially important to build capacity in reducing or controlling sources of mercury pollution as nine of the countries assessed in this study have already published Minamata Initial Assessment studies outlining national needs to enhance environmental regulations regarding industrial emissions, waste and wastewater management, and other anthropogenic sources of mercury releases that directly affect mercury inputs into fresh and coastal waters. Fish possess traits that make them excellent biosentinels for tracking mercury through time (Eagles-Smith et al. 2016; Braaten et al. 2019; Grieb et al. 2020). For example, younger, smaller, forage fish are ideal indicators for localized spatial and short temporal resolution, while larger pelagic species can integrate patterns from larger areas and across longer time frames (Mason et al. 2005). Additionally, the global ubiquity of fish consumption allows biomonitoring to contextualize and inform our understanding of risk to human populations (Sunderland 2007).

Mercury concentrations in individual fish vary between individuals and species due to a number of factors (e.g., Somers and Jackson 1993; Hammerschmidt and Fitzgerald 2006; Petre et al. 2012; Sackett et al. 2013; Eagles-Smith et al. 2018). These include individual- (e.g., age, diet, growth rate, other physiological factors) and species- (e.g., life history traits, foraging guilds) specific traits along with environmental (e.g., mercury inputs, methylation rate, site-specific food web length) and temporal context (annual and seasonal patterns) (Harris and Bodaly 1998; Wiener et al. 2003; Simoneau et al. 2005; Eagles-Smith and Ackerman 2009; Walters et al. 2010; Braaten et al. 2014). Predatory fish at higher trophic levels are among the most highly contaminated due to the biomagnification process (Hall et al. 1997; Vander Zanden and Rasmussen 1996; Sackett et al. 2013; Bastos et al. 2015; Rolfhus et al. 2011; Wu et al. 2019). Additionally, older fish are generally more contaminated than younger fish due to bioaccumulation factors (Somers and Jackson 1993; Trudel and Rasmussen 2006; Piraino and Taylor 2009; Braaten et al. 2014; Sunderland et al. 2018). However, these processes are conflated by the amount of mercury available in a given system as well as other factors, such as metabolic costs of individual organism behaviors (e.g., foraging, spawning, predator avoidance) or elevated growth rates (e.g., Harris and Bodaly 1998; Trudel and Rasmussen 2006; Van Walleghem et al. 2007; Ward et al. 2010; Dang and Wang 2012; Eagles-Smith et al. 2008, 2018). As a result, informed choices about the species, size, and location of fish can potentially influence mercury exposure in fish-consuming humans and biota in the wider Caribbean Region. In this study, we present an initial evaluation of mercury concentrations in fish and invertebrates captured in the wider Caribbean Region from 2005–2023.

## Methods

### Study Area

**Fig. 1:**
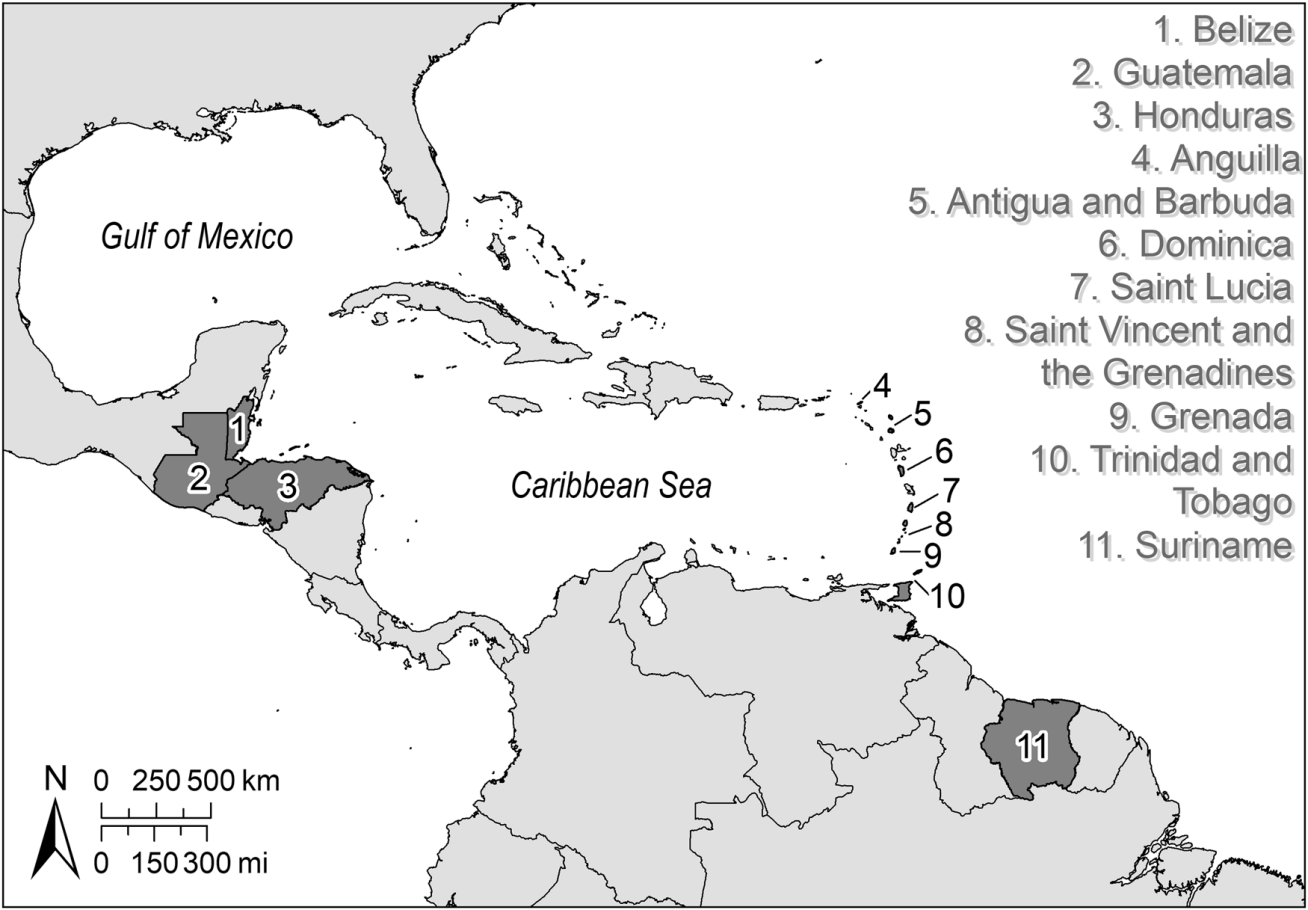
Focal countries and territories in the wider Caribbean Region

Fish and commonly consumed invertebrates were collected from eleven countries or territories in the wider Caribbean Region (Fig. 1). This assessment includes seven islands countries or territories (Anguilla, Antigua and Barbuda, Dominica, Grenada, Saint Lucia, Saint Vincent and the Grenadines, Trinidad and Tobago; referred to as the Islands hereafter) and four countries on the Latin American mainland (Belize, Honduras, Guatemala, and Suriname). Hereafter, we refer to the wider region of the Caribbean Sea, Gulf of Mexico (GOM), and the freshwater and estuarine waters of Central America and Caribbean countries as the wider Caribbean Region. Suriname was included as it is a member of the Caribbean Community (CARICOM). This region includes the Mesoamerican Reef, the largest barrier reef system in the Western Hemisphere, and a biodiversity hotspot.

### Fish and Invertebrate Sampling

Fish and invertebrate samples were collected over an 18-year period between 2005 and 2023 (Table 1). Samples were collected from local fishermen, at local fish markets, with nets, or with rod and reel. Samples were collected as part of independent pilot sampling projects and compiled here for regional analysis resulting in sample sizes varying widely between species as well as geospatially and temporally. Priority species were identified based on local availability and consumption patterns. When whole fish were available, total length (TL) was recorded and a photograph was taken for species confirmation. Individuals collected were identified at the collection site and photographs were reviewed by fisheries biologists for confirmation as needed. All samples were assigned a Taxonomic Serial Number (TSN) using the Integrated Taxonomic Information System (ITIS) based on the lowest available taxonomic level that could be reliably verified (ITIS 2023). Efforts were made to obtain approximate locations of capture for each sample, through this spatial information was not always available. Fish trophic levels were assigned by species from FishBase using the ‘rfishbase’ package within the R statistical modeling environment (Boettiger et al. 2012; Froese and Pauly 2023; R Core Team 2023). In cases where species- or genus-level identifications were not available or reliable, the mean trophic level for the genus or family was used, respectively.

**Table 1:** The number of included species and sample size by country, along with the number and range of years where sampling occurred, by country in the wider Caribbean Region

Country	Number of Species	Number of Samples	Number of Sampling Years	Range of Years
Anguilla	6	63	1	2019
Antigua and Barbuda	23	150	4	2018–2022
Belize	45	522	6	2005–2019
Dominica	11	78	1	2018
Grenada	15	87	1	2018
Guatemala	3	73	1	2010
Honduras	29	198	2	2011–2016
Saint Lucia	14	67	2	2018–2023
Saint Vincent and the Grenadines	9	40	1	2018
Suriname	6	48	1	2018
Trinidad and Tobago	23	280	3	2017–2023
**Total**	**108**	**1606**	**13**	**2005**–**2023**

Field sampling procedure were adapted from U.S. Environmental Protection Agency (U.S. EPA 2000) and U.S. Geological Survey (Scudder et al. 2008). A muscle sample from individual fish (skin-off fillet) or edible tissue from invertebrates was taken and placed in resealable plastic bags for storage. All samples were kept on ice in the field and then immediately transferred to a −20 °C freezer for storage prior to shipment to the laboratory. Samples were subsequently shipped to Biodiversity Research Institute (BRI) Toxicology Laboratory in Portland, Maine, USA, in coolers with freezer packs or dry ice using expedited delivery and all appropriate export and import documents (e.g., U.S. Fish and Wildlife Service Form 3–177) were obtained.

### Laboratory analysis

All fish and invertebrate tissue samples were analyzed at the BRI Toxicology Laboratory. Single tissue samples from each individual were analyzed as wet weight using EPA Method 7473 (U.S. EPA 1998) by gold-amalgamation atomic absorption spectroscopy following thermal desorption of the sample using either a Milestone DMA-80 or a Nippon MA-3000. The equipment was evaluated following calibration. Subsequently, the equipment performance was checked at the beginning and end of each run and after every 10 samples using two certified reference materials (NRC DOLT-4 and ERM CE-464) and check blanks. Recovery of reference materials was confirmed to be within 10% of certified values. The instrument detection limit (IDL) is 0.001 µg/g.

### Statistical analysis

For all data, we present the geometric mean, unless otherwise noted, as the estimate of central tendency because of the non-normal distribution of the data. All mercury concentration data was natural log transformed before statistical analyses to meet the assumptions of parametric tests. Results are presented as µg/g ww, unless otherwise noted, allowing for direct comparison with U.S. FDA/EPA guidelines for human consumption to aid in interpretation (U.S. FDA/EPA 2022). Data are first presented as non-standardized THg concentrations, rather than after standardization (e.g., length, age), unless otherwise noted, in order to better understand the context of individual THg concentrations in fish and invertebrates being consumed by humans and biota even through it confounds interpretation of the mechanisms underlaying observed patterns.

Comparisons of mercury concentrations between freshwater and marine systems are complicated by differing life-history traits. Growth rate disparities can mean smaller freshwater individuals of some species can be older than a similar or larger fish of a marine species (e.g., Winemiller and Rose 1991). As a result, we evaluated patterns in mercury concentrations between freshwater and saltwater fish both as non-standardized THg concentrations and after correcting for age using an ANCOVA analysis with estimated age and habitat. Age was estimated from length using growth parameters extracted from the ‘rfishbase’ package (Boettiger et al. 2012; R Core Team 2023). In cases where species- or genus-level parameters were not available, the mean for the genus or family was used, respectively.

In order to further explore patterns in the mercury concentrations observed in fish, a mixed effects model was used to account for confounding factors. Only fish with a known length (n = 1351) were included in the model analysis and invertebrate samples were excluded. The response variable was natural log transformed THg concentrations, and was transformed to improve normality before analysis. The model was fit using the ‘lme4’ and ‘emmeans’ packages (Bates et al. 2015; Lenth 2023; R Core Team 2023). The final model was parametrized to explore spatial patterns between countries in the wider Caribbean Region as well as between taxonomic family groups. In the final model, taxonomic family, country of sampling, trophic level, and fish length were included as fixed effects along with year, considered as a factor, as a random effect. Model fit was evaluated using marginal and conditional R^2^ values. Patterns in model residuals were also examined to understand model performance.

## Results

Between 2005 and 2023, we analyzed 1606 samples, including 52 invertebrate samples, for THg from the wider Caribbean Region. There were 108 unique species (103 fish and five invertebrate species) sampled from 47 families. Marine and coastal estuarine samples accounted for 1211 samples (including all 52 invertebrates), while freshwater fish accounted for the remaining 395 samples. The overall geometric mean across all samples was 0.19 µg/g ww [min, max: 0.001, 5.71].

### Mercury concentrations and human health guidelines

Consumption of fish with elevated concentrations of mercury has been linked to adverse impacts on human health (Karagas et al. 2012; U.S. FDA/EPA 2022). The U.S. FDA/EPA publishes consumption guidelines for sensitive populations (those who are or might become pregnant, are breastfeeding, and children ages 1–11) based on mercury concentrations in fish muscle (U.S. FDA/EPA 2022). Across all species and locations, 681 (42.4%) individuals were equal to or below the 0.15 µg/g ww threshold that classifies best choices (three weekly servings). A further 201 (12.5%) and 302 (18.8%) had concentrations of 0.15–0.23 µg/g ww (two weekly servings) and 0.23–0.46 µg/g ww (one weekly serving), respectively, that are both classified as good choices. Finally, 422 (26.3%) individuals exceeded the 0.46 µg/g ww threshold that the U.S. FDA / EPA classifies as choices to avoid (zero weekly servings).

### Mercury concentrations by family

**Fig. 2:**
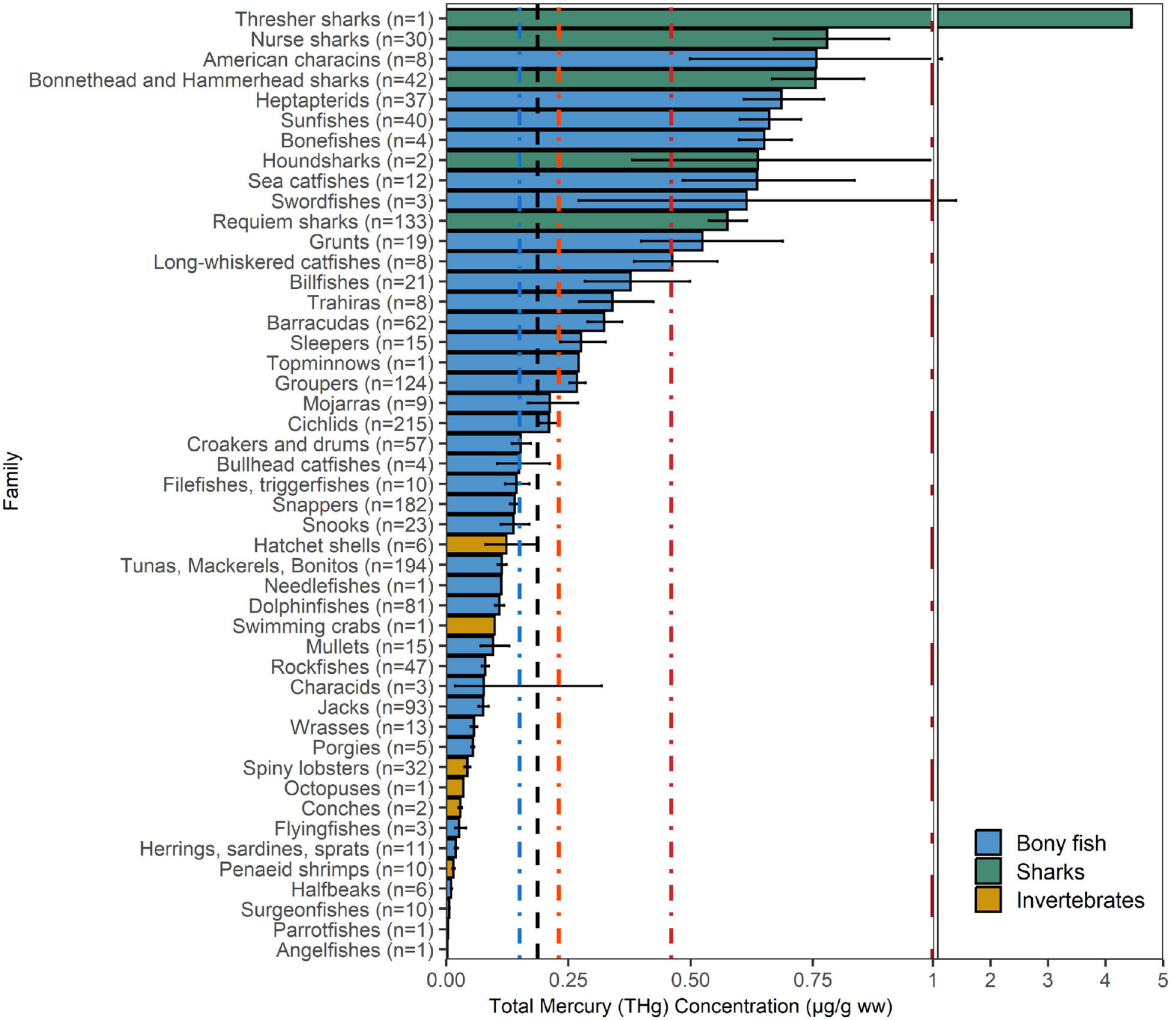
Fish muscle and invertebrate THg concentrations (µg/g ww) by taxonomic family (bars) in the wider Caribbean Region. Bars are geometric means and error bars are standard errors. The black dashed vertical line represents the overall geometric mean THg sampled in the wider Caribbean Region. The colored dashed vertical reference lines are the U.S. FDA/EPA (2022) fish consumption guidelines for sensitive human populations of 0.15 (blue), 0.23 (orange), and 0.46 (red) μg/g ww in fish muscle indicating best choices ( ≤ 0.15 = 3 weekly fish servings), good choices (≤0.23 = 2 weekly fish servings or ≤0.46 = 1 weekly fish serving), or choices to avoid (>0.46 = 0 weekly fish servings) (U.S. FDA/EPA 2022) as well as the WHO threshold for some predatory fish (1.0 μg/g ww, brown) (U.S. FDA/EPA 2022; FAO/WHO 2007)

Mercury concentrations varied significantly by taxonomic family (*F*_*46,1559*_ = 28.94, p < 0.001; Fig. 2). Overall, 25 families had a mean THg concentration below the U.S. FDA/EPA guideline threshold of 0.15 µg/g ww in muscle. Surgeonfishes (Acanthuridae; 0.006) [0.004, 0.012] µg/g ww; n = 10; trophic Level=2.27 were the only family with more than one sampled individual and a mean THg concentration below 0.01 µg/g ww; though single individuals were sampled from Angelfishes (Pomacanthidae; 0.003 µg/g ww; n = 1; trophic Level=2.80) and Parrotfishes (Scaridae; 0.004 µg/g ww; n = 1; trophic Level=2.03) that were below this value. Twelve families (as well as the one thresher shark sampled) had a mean THg concentrations in muscle greater than the U.S. FDA/EPA guidelines limit of 0.46 µg/g ww and tended to occupy higher trophic levels. Five of these 13 families were sharks. The other fish families above 0.46 µg/g ww include swordfishes (Xiphiidae; 0.61 [0.16, 2.73] µg/g ww; n = 3; trophic Level=4.53), bonefishes (Albulidae; 0.65 [0.53, 0.75] µg/g ww; n = 4; trophic Level=3.29), and American characins (Characidae; 0.76 [0.07, 2.40] µg/g ww; n = 8; trophic Level=2.99).

### Caribbean region mercury monitoring network (CRMMN) target species

**Fig. 3:**
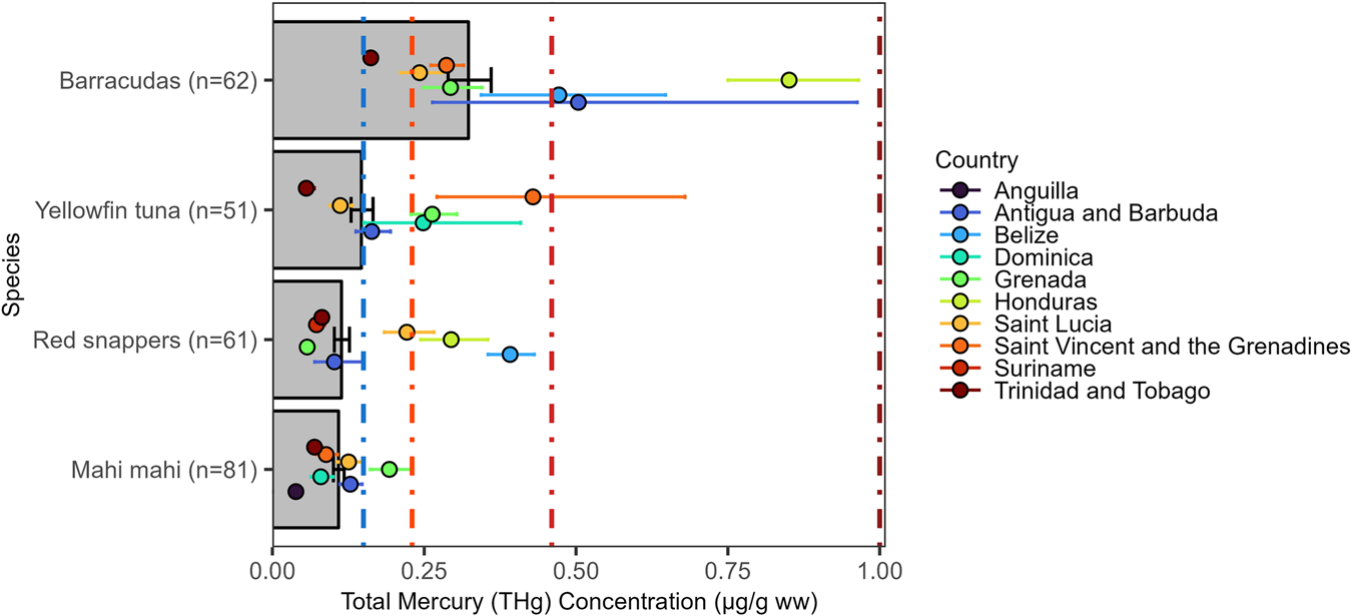
Target species muscle Total Mercury (THg) concentrations (µg/g ww) by species (bars) in the wider Caribbean Region. Bars are geometric means and error bars are standard errors. The colored dashed vertical reference lines are fish consumption guidelines (see Fig. 2 for details)

CRMMN target species were identified as barracudas (*Sphyraena sp*.), mahi mahi (*Coryphaena hippurus*), red snappers (*Lutjanus campechanus* and *L. purpureus*), and yellowfin tuna (*Thunnus albacares*) (Table 2). Barracudas had the highest mean THg concentration of 0.32 [0.03, 2.53] µg/g ww followed by yellowfin tuna at 0.15 [0.01, 0.68] µg/g ww (Fig. 3). Red snappers (0.11 [0.03, 0.64] µg/g ww) and mahi mahi (0.11 [0.01, 0.47] µg/g ww) had lower mean THg concentrations.

**Table 2:** Sample sizes by country for the four Caribbean Mercury Monitoring Network (CRMMN) target species: Barracudas (*Sphyraena sp*.), mahi mahi (*Coryphaena hippurus*), red snappers (*Lutjanus campechanus* and *L. purpureus*), and yellowfin tuna (*Thunnus albacares*)

Country	Barracudas	Mahi mahi	Red snappers	Yellowfin tuna
Anguilla	–	1	–	–
Antigua and Barbuda	3	32	6	19
Belize	16	–	9	–
Dominica	–	10	–	5
Grenada	7	8	5	8
Honduras	5	–	2	–
Saint Lucia	13	10	5	7
Saint Vincent and the Grenadines	8	8	–	2
Suriname	–	–	8	–
Trinidad and Tobago	10	12	26	10
**Total**	**62**	**81**	**61**	**51**

### Invertebrates

**Fig. 4:**
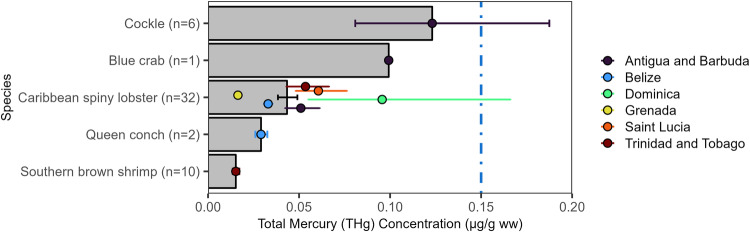
Invertebrate muscle Total Mercury (THg) concentrations (µg/g ww) by country (bars) in the wider Caribbean Region. Bars are geometric means and error bars are standard errors. The colored dashed vertical reference lines are fish consumption guidelines (see Fig. 2 for details)

In total, THg concentrations were analyzed in 51 invertebrate samples from five species that could be identified to species. Overall, THg concentrations were low with a mean of 0.04 [0.01, 0.69] µg/g ww (Fig. 4). THg concentrations were highest in six samples of cockle (0.12 [0.04, 0.45] µg/g ww). Three (5.9%) of these invertebrate samples exceeded 0.15 µg/g ww, the lowest U.S. FDA/EPA threshold.

Caribbean spiny lobster (*Panulirus argus*) was the only invertebrate species sampled in more than one country. THg concentrations were highest in Dominica (0.10 [0.04, 0.69] µg/g ww; n = 5) and lowest in Grenada (0.02 [0.02, 0.02] µg/g ww; n = 2). One sample from Dominica exceeded the 0.46 µg/g ww U.S. FDA/EPA threshold to avoid for sensitive populations (U.S. FDA/EPA 2022). The remaining 31 samples were all below the lowest U.S. FDA/EPA threshold. There were significant differences found between the Caribbean spiny lobster that were sampled in the different countries in the wider Caribbean Region (*F*_*5,26*_ = 4.113, p = 0.0069). Caribbean spiny lobster THg concentrations in Dominica were significantly higher than in Belize and Grenada, but there were no other significant differences observed.

### Lionfish (Pterois volitans)

**Fig. 5:**
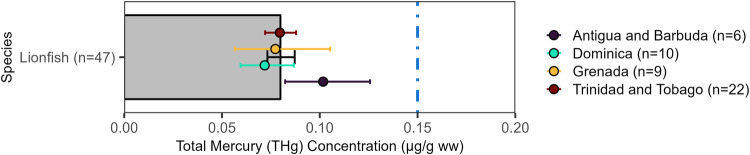
Lionfish (*Pterois volitans*) muscle Total Mercury (THg) concentrations (µg/g ww) by country (points) in the wider Caribbean Region. Bars and points are geometric means and error bars are standard errors. The colored dashed vertical reference lines are fish consumption guidelines (see Fig. 2 for details)

Lionfish (*Pterois volitans*; trophic Level=4.35) had an overall mean THg of 0.08 [0.02, 0.50] µg/g ww (Fig. 5). They were sampled in four countries in the wider Caribbean Region, including Antigua and Barbuda (n = 6), Trinidad and Tobago (n = 22), Grenada (n = 9), and Dominica (n = 10). The lowest levels were found in Dominica (0.07 [0.035, 0.23] µg/g ww) and the highest in Antigua and Barbuda (0.10 [0.04, 0.19] µg/g ww; n = 6), although there was no significant difference between lionfish sampled from these countries (*F*_*3,43*_ = 0.424, p = 0.74).

### Croakers and drums (Sciaenidae)

**Fig. 6:**
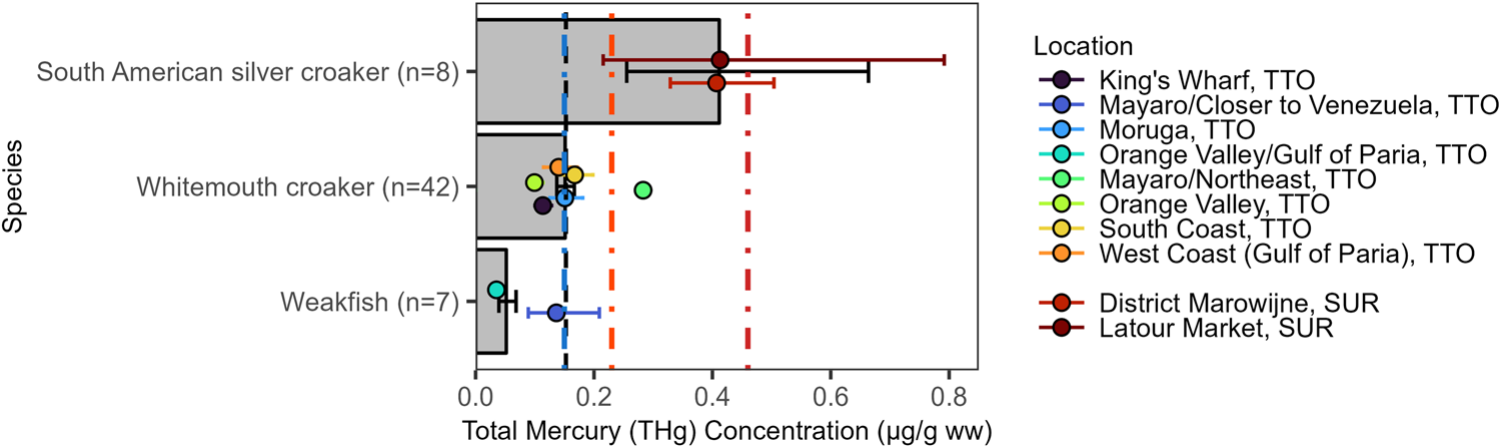
Croaker and drum (Sciaenidae) muscle Total Mercury (THg) concentrations (µg/g ww) by species (bars) and best available capture location (points) in the wider Caribbean Region. Bars and points are geometric means and error bars are standard errors. The black dashed vertical line represents the overall geometric mean THg sampled in the wider Caribbean Region. The colored dashed vertical reference lines are fish consumption guidelines (see Fig. 2 for details)

We sampled 57 individuals from three species of the family Sciaenidae, including South American silver croaker (*Plagioscion squamosissimus*; n = 8; trophic Level=4.35), whitemouth croaker (*Micropogonias furnieri*; n = 42; trophic Level=3.09), and weakfish (*Cynoscion acoupa;* n = 7; trophic Level=4.05). The overall mean THg concentration was 0.15 [0.02, 1.22] µg/g ww (Fig. 6). South American silver croaker had the highest overall mean THg concentration of 0.41 [0.02, 1.22] µg/g ww of the species in the family included in this study and all eight were collected in Suriname.

### Grunts (Haemulidae)

**Fig. 7:**
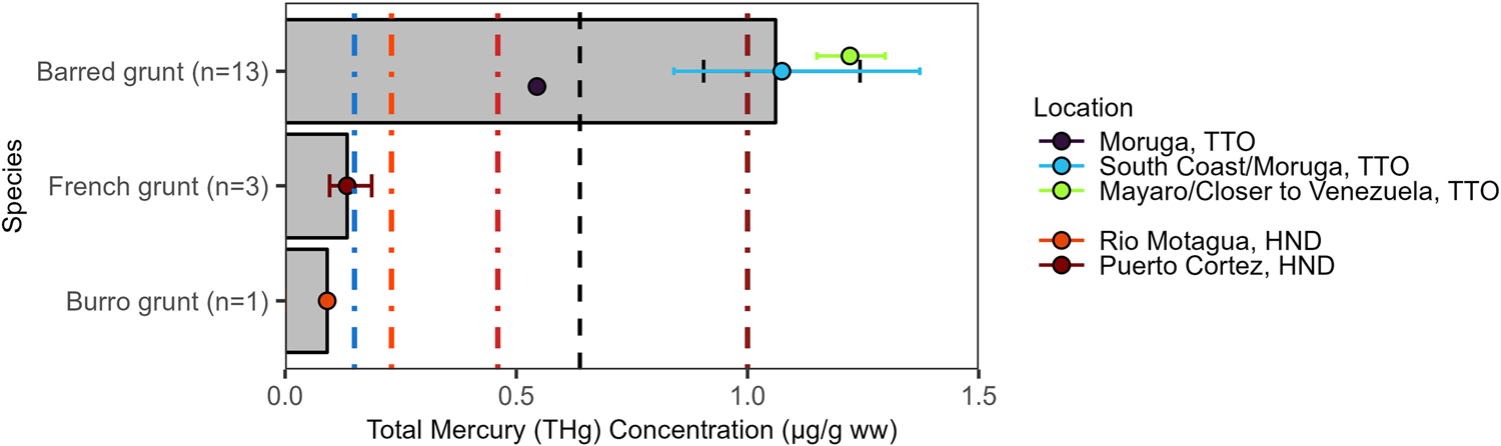
Grunt (Haemulidae) muscle Total Mercury (THg) concentrations (µg/g ww) by species (bars) and best available capture location (points) in the wider Caribbean Region. Bars and points are geometric means and error bars are standard errors. The black dashed vertical line represents the overall geometric mean THg sampled in the wider Caribbean Region. The colored dashed vertical reference lines are fish consumption guidelines (see Fig. 2 for details)

In total, we sampled 17 individuals that could be identified as three species (two additional individuals identified as *Haemulon sp*. were not included in results in this section) in the Haemulidae family with an overall mean THg concentration of 0.64 [0.08, 2.34] µg/g ww, which exceeds the U.S. FDA/EPA threshold recommending no consumption for sensitive populations (Fig. 7). The barred grunt (*Conodon nobilis*) had an overall mean of 1.06 [0.34, 2.34] μg/g ww (n = 13; trophic level = 3.61) that was elevated compared to the French grunt (*Haemulon flavolineatum*; 0.13 [0.08, 0.25] μg/g ww; n = 3; trophic Level=3.46) and burro grunt (*Pomadasys crocro*; 0.09 μg/g ww; n = 1; trophic Level=4.03). All thirteen barred grunts were collected in Moruga or Mayaro, Trinidad and Tobago and all were captured off the south coast between Trinidad and Venezuela. Only one individual did not exceed the 0.46 μg/g ww U.S. FDA/EPA guideline for choices to avoid.

### Groupers (Serranidae)

**Fig. 8:**
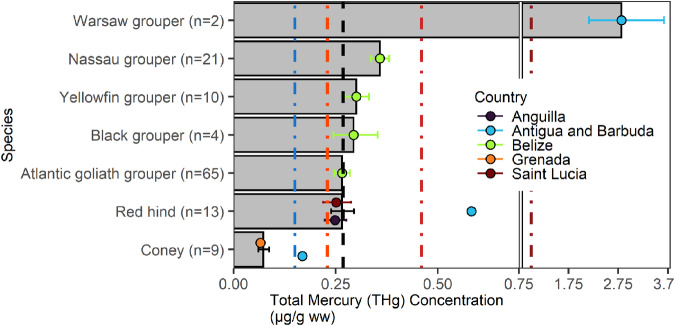
Groupers (Serranidae) muscle Total Mercury (THg) concentrations (µg/g ww) by species (bars) in the wider Caribbean Region. Bars are geometric means and error bars are standard errors. The black dashed vertical line represents the overall geometric mean THg sampled in the wider Caribbean Region. The colored dashed vertical reference lines are fish consumption guidelines (see Fig. 2 for details)

Between 2006 and 2023, 124 grouper individuals were sampled from seven species (Fig. 8). The overall mean THg concentration was 0.27 [0.03, 3.67] µg/g ww. Warsaw grouper (*Hyporthodus nigritus*) had the highest geometric mean THg concentration (2.82 [2.16, 3.67] μg/g ww; n = 2; trophic Level=3.97), well above the U.S. FDA/EPA threshold to avoid for sensitive populations (U.S. FDA/EPA 2022).

### Sharks (Elasmobranchii)

**Fig. 9:**
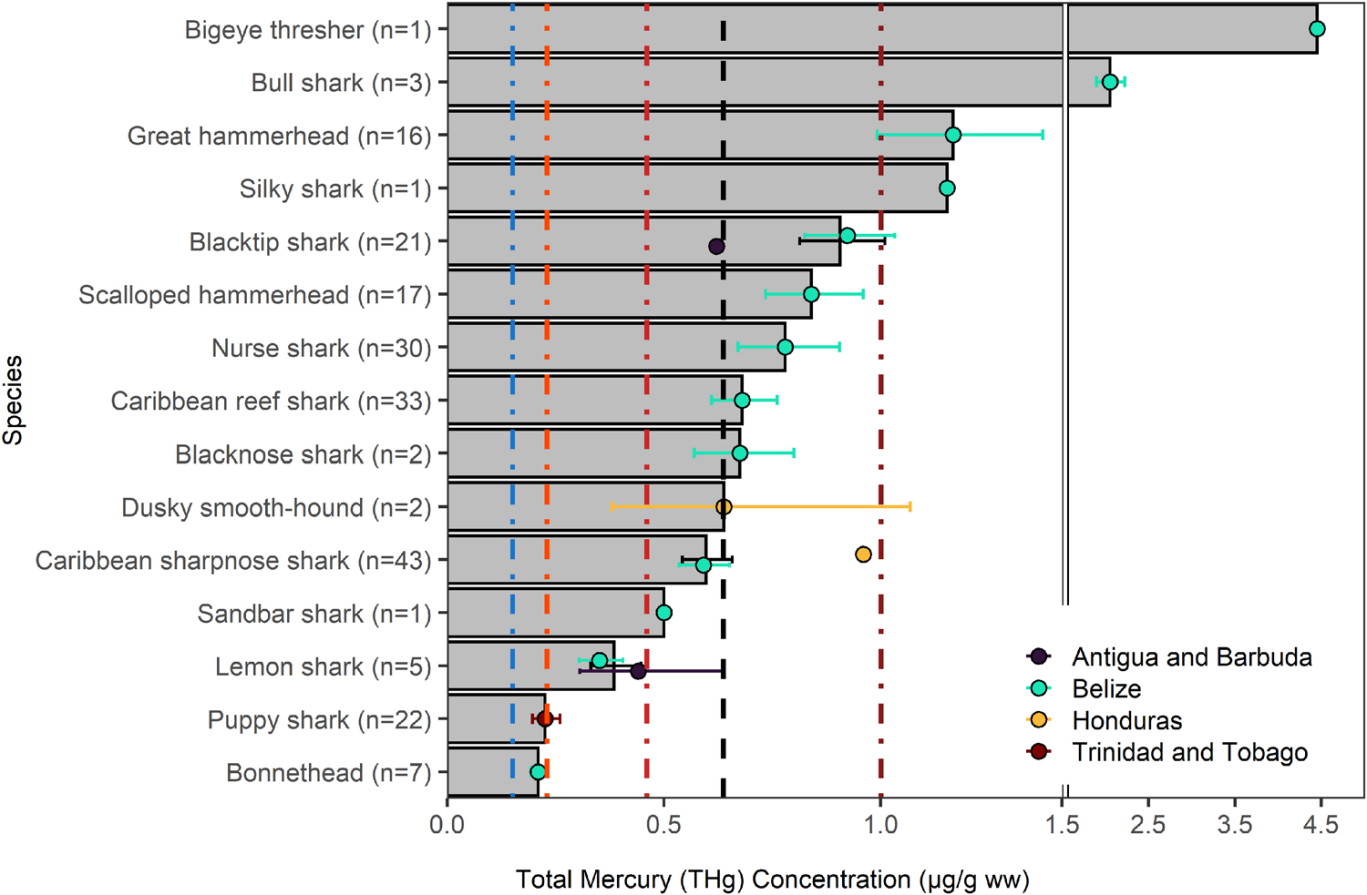
Shark muscle Total Mercury (THg) concentrations (µg/g ww) by species (bars) and country (points) in the wider Caribbean Region. Bars and points are geometric means and error bars are standard errors. The black dashed vertical line represents the overall geometric mean THg sampled in the wider Caribbean Region. The colored dashed vertical reference lines are fish consumption guidelines (see Fig. 2 for details)

In total, 204 shark samples were analyzed. The overall mean THg in shark muscle in the wider Caribbean Region was 0.64 [0.06, 4.46] µg/g ww, which exceed the U.S. FDA/EPA threshold recommending no consumption for sensitive populations (Fig. 9). Mean THg concentrations for the different shark species were lowest in bonnetheads (*Sphyrna tiburo*; 0.21 [0.16, 0.27] µg/g ww; n = 7; trophic Level=4.0) and puppy sharks (*Carcharhinus porosus*; 0.23 [0.06, 0.84] µg/g ww, n = 22; trophic Level=4.14). The THg concentrations were highest in bigeye threshers (*Alopias superciliosus*; 4.45; n = 1; trophic Level=4.46) and bull sharks (*Carcharhinus leucas*; 2.06 [1.76, 2.29] µg/g ww; n = 3; trophic Level=4.31). The mean THg concentration in 12 of 15 shark species (as well as the overall geometric mean for all shark individuals) and 68% of individuals had THg concentrations that exceeded 0.46 µg/g ww, the threshold above which the U.S. FDA/EPA (2022) guidelines recommend avoiding consumption for sensitive populations.

### Saltwater and freshwater habitats

**Fig. 10:**
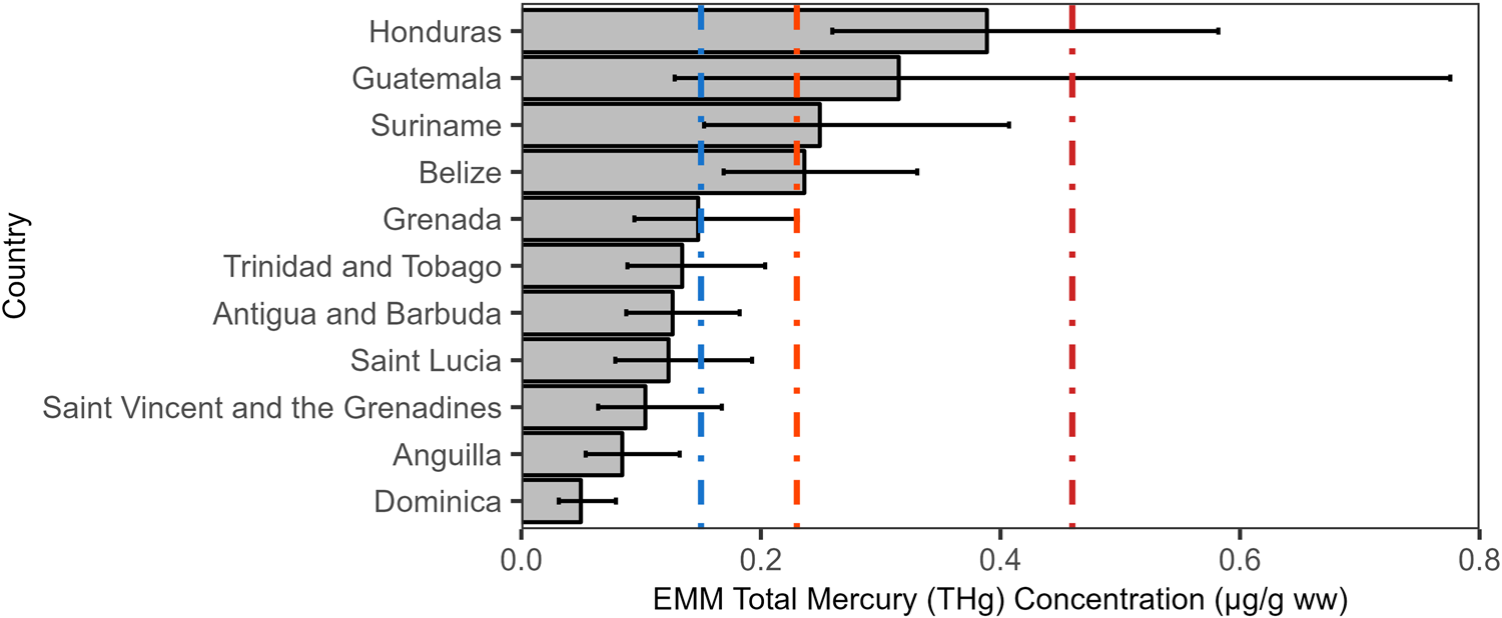
Estimated marginal mean of total mercury concentration in fish muscle (μg/g ww) by country after accounting for taxonomic family, fish length, trophic level, and sampling year. Error bars are 95% confidence intervals around the mean. The colored dashed vertical reference lines are fish consumption guidelines (see Fig. 2 for details)

The fish sampled from the wider Caribbean Region originated in both freshwater and marine ecosystems. The mean THg concentration was significantly higher in freshwater systems (0.29 [0.01, 3.72] µg/g ww; n = 395) than marine (0.17 [0.00, 5.71] µg/g ww; n = 1159) systems (t = 7.1, df=578.6, p < 0.001) (Fig. 10). This pattern was first examined before size-standardization due to the desire to understand THg concentrations in actual individuals available for consumption within the region. The significant relationship between habitats was maintained even after accounting for estimated fish age (ANCOVA; F_1,1426_ = 59.866; p < 0.001). There was no significant relationship found between THg and estimated age after accounting for habitat (ANCOVA; F_1,1426_ = 0.437; p = 0.508).

### Modeling mercury concentrations in the Caribbean region

We used a mixed effects model to further explore the variability in fish mercury concentrations in the wider Caribbean Region. The overall model fit was good (conditional R^2^ = 0.64) and the fixed effects described below were important in predicting THg concentrations (marginal R^2^ = 0.56). The final model had a root mean square error (RMSE) of 0.77 and the residual and actual versus predicted THg concentration plots are presented in the supplementary information (Fig. S1 and S2). Taxonomic family, country, trophic level, and length were all found to have significant relationships with THg (p < 0.001) for samples where these variables were all available.

Using the model to account for covariates, we found significant differences between the countries where sampling was conducted (p < 0.001; Fig. 10). Four countries – Honduras, Guatemala, Suriname, and Belize—had model predicted means that exceeded the 0.23 µg/g ww guideline. The remaining countries were below the lowest threshold. There were also significant differences found between families with bonefishes (Albulidae), grunts (Haemulidae), American characins (Characidae), and Heptapterids (Heptapteridae) exceeding 0.46 µg/g ww guideline for choices to avoid (p < 0.001). Similarly, we found significant relationships between predicted THg concentrations in fish for both fish length and trophic level (p < 0.001).

## Discussion

The fate of mercury in the environment is complex and its bioaccumulation and biomagnification in a system is confounded by a number of factors. As a result, the threat of mercury exposure is not homogenously distributed across the landscape or between species and individuals. Biomonitoring, temporally and spatially, can inform our understanding of the individual, spatial, and taxonomical drivers in the magnitude of mercury concentrations.

We analyzed a dataset of more than 1600 fish and aquatic invertebrate samples from the wider Caribbean Region collected between 2005 and 2023. Overall, the magnitude of THg concentrations was relatively low, with 55% of all samples under the U.S. FDA/EPA 0.23 µg/g ww threshold for vulnerable populations. Arithmetically, the four mainland countries had the highest mean mercury concentrations after accounting for taxonomic family, length, trophic level, and sampling year. These results indicate that spatial patterns in mercury inputs into the environment and in ecosystem sensitivity—the methylation potential—may both be playing a role in the THg concentrations in the wider Caribbean Region. Additionally, there was considerable taxonomic and between individual variation that largely match our a priori understanding of how mercury moves through systems.

### Human health

Fisheries, particularly small-scale, play an important cultural and economic role in the wider Caribbean Region (Girvan 2021). However, the overall mean consumption of aquatic food in Latin America and the Caribbean (9.9 kg capita^−1^ yr^−1^) is well below the global average (20.5 kg capita^−1^ yr^−1^) in estimates for 2019 (FAO 2022). However, consumption rates vary broadly by country within the wider Caribbean Region, from 2 kg in Belize to as high as 59 kg in Antigua and Barbuda (CRFM 2021; FAO 2022). As a result, the consumption of aquatic products in the wider Caribbean Region is expected to continue increasing, particularly consumption of regionally sourced aquatic food (Girvan 2021; CRFM 2021; FAO 2022). This expected trend highlights the importance of understanding patterns in THg concentrations in fish and other seafood, the primary route of human exposure to mercury, and subsequent potential toxicological impacts of mercury on biota and human health (Sunderland 2007).

There are well established thresholds in published literature that can be used to assess mercury concentrations and the adverse impacts of consumption of fish and invertebrates with elevated levels of mercury (U.S. FDA/EPA 2022). Overall, 42.4% of all samples were classified as a ‘best choice’; however, 26% were classified as ‘choices to avoid’ by the U.S. FDA/EPA (2022) guidelines. Further analyses indicated that there was considerable variation in the magnitude of THg concentrations, which were significantly impacted by a suite of covariates. As a result, understanding spatial and taxonomic patterns in the magnitude of mercury concentrations will be increasingly important in the wider Caribbean Region.

### Spatial drivers in mercury concentrations

The overlapping and conflating factors that impact THg concentrations in fish make interpretation complex and understanding of the mechanisms driving spatial patterns challenging to parse. However, past studies have also found geospatial patterns in mercury concentrations in seafood (e.g., Sunderland et al. 2018). Mercury exposure can be attributed to both the amount of mercury that is entering a system (e.g., releases from a point source, atmospheric deposition) or, alternatively, the potential for the mercury to be methylated into its bioavailable form – MeHg – a highly heterogenous process that can decouple mercury concentrations in biota from inorganic mercury inputs (Hsu-Kim et al. 2013, 2018; Shanley et al. 2012; Kocman et al. 2017; Travnikov et al. 2017; Grieb et al. 2020; Richter and Skinner 2020). Both anthropogenic and natural events can mobilize mercury through processes (e.g., erosion, sedimentation, recirculation) that are increasingly important with the increase in extreme events associated with climate change (Balogh et al. 1998; Roulet et al. 2000; Outridge et al. 2018; Diringer et al. 2020; Liu et al. 2021).

Our analysis indicates that mean predicted THg concentrations were arithmetically higher in the four countries on the Latin American mainland after accounting for the other covariates (taxonomic family, trophic level, size, sampling year). However, the lack of consistently available geospatial data makes discerning the drivers of these spatial patterns challenging. Notably, the fish from four mainland countries may be subject to higher mercury inputs to their ecosystems due a variety of factors, including industrial activities, consumer waste, and ASGM (Rojas de Astudillo et al. 2005; Kocman et al. 2017; UNEP 2019; Government of Suriname 2020; Liu et al. 2021). These countries generally had higher rates of total mercury emissions and releases according to the available Mercury Initial Assessments (MIAs; Mainland: Belize, Guatemala, and Suriname; Islands: Antigua and Barbuda, Dominica, Grenada, Saint Lucia, Saint Vincent and the Grenadines, and Trinidad and Tobago) with notable outliers in Suriname and Trinidad and Tobago. The average total emissions and releases from the Islands was 823 kg Hg yr^−1^ (39.6 kg Hg yr^−1^ excluding Trinidad and Tobago) compared to 31,434 kg Hg yr^−1^ (2719 kg Hg yr^−1^ excluding Suriname) in the mainland countries.

ASGM is now the leading source of global mercury emissions, particularly in the Global South, and is associated with a suite of ecological impacts (e.g., deforestation, erosion) that directly and indirectly impact mercury fate and transport (Roulet et al. 2000; Swenson et al. 2011; Asner et al. 2013; Caballero Espejo et al. 2018; UNEP 2019; Diringer et al. 2020). Suriname reports more than 62,000 out of a total of 88,864 kg Hg yr^−1^ in emissions and releases from ASGM, with 23,829 kg Hg yr^−1^ estimated to be released into aquatic systems (Government of Suriname 2020). These emissions and releases from ASGM alone are nearly six times the combined total from the other eight countries with available MIAs. No other countries in this analysis with available MIAs reported any ASGM activity, though there is some evidence it is occurring in other countries in the region, including Honduras and Guatemala (AMAP/UNEP 2019; Yoshimura et al. 2021). The mean freshwater fish THg concentration in Suriname (0.48 [0.02, 2.40] µg/g ww; n = 40) was the highest in this study. The overall geometric mean of South American silver croakers, a freshwater Sciaenidae (croakers and drums) species captured in Surinamese rivers, was 0.41 [0.02, 1.22] µg/g ww. In fact, all five freshwater fish species sampled in Suriname had mean THg concentrations exceeding 0.23 µg/g ww with the lowest mean concentration seen in anjumara (*Hoplias aimara*; 0.34 [0.12, 0.78] µg/g ww; n = 8; trophic Level=3.74) and the highest in redeye piranha (*Serrasalmus rhombeus*; 0.76 [0.07, 2.40] µg/g ww; n = 8; trophic Level=4.03). However, examining the model residuals found that Suriname had the arithmetically largest, positive residuals indicating underprediction of THg concentrations and supporting the need for further sampling.

Other source sectors that are non-uniformly distributed in the wider Caribbean Region include industrial processes and consumer products. The mainland countries with available MIAs had mean emissions and releases from consumer products of 599 kg Hg yr^−1^ compared to 52 kg Hg yr^−1^ for Island states. This pattern is corroborated at a more local level by a recent study of Atlantic goliath grouper that found that, while overall mercury concentrations were high, the highest levels were found near urban runoff, indicating that spatial patterns in emissions and releases can impact mercury concentrations in the wider Caribbean Region (Malinowski 2019). At three sites in Florida, Adams et al. (2003) also found considerable inter- and intra-site variability in mean THg concentrations of 1.15 [0.09, 3.30], 0.13 [0.01,0.58], and 0.35 [0.10, 0.65] µg/g ww for mean lengths of 40.7, 132.0, and 54.0 cm standard length, respectively; while Malinowski (2019) found a mean THg of 1.21 [0.21, 7.60] and 0.47 [0.16, 0.94] µg/g ww for adults (>110 cm TL) and juvenile individuals (<110; mean=70.6 cm TL), respectively.

The wider Caribbean Region has multiple river systems that input an estimated total of 105 Mg Hg yr^−1^ into coastal waters sourced to a number of anthropogenic inputs (Liu et al. 2021). There are also multiple river basins in northern South America that discharge into the Atlantic Ocean where the Guiana Current circulates ocean water along the coast towards Trinidad. The South Guiana Coast – Amazon Cone Basin alone is estimated to discharge 120 Mg Hg yr^−1^ (Liu et al. 2021). More locally, Trinidad and Tobago and Venezuela both have oil and gas production in addition to other heavy industry that contribute runoff to the waters between the two countries with some evidence that proximity to industrial sites is linked to high metal concentrations in marine sediments in the region (Rojas de Astudillo 2005). Trinidad and Tobago reported the highest levels of total mercury emissions and releases from the fuel/energy sector (3654 kg Hg yr^−1^) of any country in this analysis with available MIA data as well as having high total emissions and releases (4741 kg Hg yr^−1^) compared to other Islands (Saint Lucia was second highest Island at a reported total of 75 kg Hg yr^−1^). Thirteen barred grunt captured southeast of Trinidad had elevated levels (1.06 [0.34, 2.34] µg/g ww; n = 13; trophic Level=3.61) compared to samples within the same taxonomic family from other locations (Fig. 7). These concentrations are close to or exceed the highest WHO guidelines for human consumption of some predatory fish (FAO/WHO 2007). Notably, according to examination of the residuals, the model underpredicted grunt (Haemulidae) THg concentrations, and particularly barred grunt sampled from Trinidad and Tobago, suggesting that other factors are contributing to measured THg concentrations. The elevated THg concentrations in these samples from south of Trinidad warrant further research, with precise geospatial data, targeting the area between Trinidad and Venezuela to better understand patterns and processes that influenced those findings.

In addition to mercury inputs, spatial variability in ecosystem sensitivity can impact the concentrations found in fish and invertebrate tissues and is driven by a variety of factors (Roué-Legall et al. 2005; Bodaly et al. 2007; Shanley et al. 2012; Crespo-Lopez et al. 2021; Gerson et al. 2022). Patterns and trends in inorganic mercury loads do not always mirror concentrations in biota (Grieb et al. 2020; Richter and Skinner 2020). While methylation rates under some conditions mean that there is a relatively large amount of bioavailable MeHg despite low inorganic mercury input, other areas may have relatively low amounts of bioavailable methylmercury despite relatively high amount of inorganic mercury inputs (Roué-Legall et al. 2005; Wyn et al. 2010). Estuarine areas, particularly mangroves, are sensitive to mercury inputs because the environments are highly conducive to mercury methylation (Podar et al. 2015; Obrist et al. 2018). Additionally, these areas are more likely exposed to higher levels of runoff (e.g., nutrients, total or dissolved organic carbon, sediment) as well as direct and legacy mercury inputs (e.g., ASGM inputs, erosion of previously contaminated soils) that impact the fate of mercury in a system in complicated ways (Chen et al. 2009, 2021; Driscoll et al. 2012; Eagles-Smith et al. 2018; Braaten et al. 2018; Taylor et al. 2019; Liu et al. 2021). These distinctions are further complicated by a number of estuarine and mangrove species, such as some species of groupers (Serranidae), that occupy multiple spatial locations and habitats through time. For example, Atlantic goliath grouper (*Epinephelus itajara*) utilize estuaries and mangroves as juveniles before moving to offshore reefs as sub-adults and adults and have high site fidelity (Koenig et al. 2007; Malinowski 2019). Atlantic goliath grouper are estimated to move out to these offshore reefs at approximately 100 cm (or 5–6 years of age) (Koenig et al. 2007). We found a significant positive relationship between THg and length (length range = 44.2–200.5 cm) in Atlantic goliath grouper (*F*_*1,54*_ = 92.16, adj. R^2^ = 0.62, p < 0.001) indicating that bioaccumulation of mercury is occurring and larger individuals may also be foraging at a higher trophic level. The analysis also indicates that this species is reaching the U.S. FDA/EPA (2022) guidelines for human consumption for sensitive population while still in coastal estuaries and mangroves. The simple linear regression model estimates Atlantic goliath grouper will surpass the 0.23 µg/g ww threshold at 61.2 cm and 0.46 µg/g ww at 102.3 cm in length. These elevated levels in smaller, estuarine and mangrove-dependent individuals can be attributed to several factors that are difficult to disentangle without further research.

Overall, the model found significant effects of THg concentrations in the wider Caribbean Region from sampling country. However, there are many confounding factors that should not be overlooked. We were only able to reliably use the country of sampling as spatial data. While trophic level was found to have a significant effect on THg concentration patterns, it was not determined for individuals in this study so was assigned using FishBase data (Froese and Pauly 2023). As a result, individuals from a given species may be feeding at a higher or lower trophic level in different spatial locations due to prey availability or characteristics, biogeochemistry, productivity, or other factors (e.g., Lavoie et al. 2013; Eagles-Smith et al. 2018). Growth and metabolism rates can also vary spatially (e.g., Eagles-Smith et al. 2018). Finally, examination of the model residuals indicate that model predictions were generally slightly better for samples from countries with larger sample sizes.

The elevated concentrations of THg in some freshwater and coastal species along with patterns in the model residuals warrants further research to better understand spatial patterns in the wider Caribbean Region and to inform if certain areas or rivers are hotspots of mercury contamination that could have potential adverse impacts on human and biotic health.

### Taxonomic drivers of mercury concentrations

Intra- and inter-species variability in THg concentrations between individuals can be attributed to multiple mechanisms, including differences in length and site-specific trophic position as described above. Using taxonomic differences allows for a generalized exploration of a suite of variables (e.g., behavioral, physiological, and reproductive) that are not always readily available and make it possible to explore patterns between groupings.

Caribbean spiny lobster (*Panulirus argus*) has long been exploited as a food source in the wider Caribbean Region, dating to pre-Colombian times, with increasing exploitation rates since the 1990s and landings >31,000 t in 2013 (Spanier et al. 2015; Pereira and Josupeit 2017; Tewfik et al. 2020). They are predatory invertebrates but the trophic chain is relatively short. They are also a prey item for many species in the wider Caribbean Region, including groupers, snappers, and sharks. As a result, Caribbean spiny lobster THg concentrations impact THg exposure in the humans and biota that consume them.

Lionfish (*Pterois volitans*) are an instructive case study illustrating the potential impacts of physiology as a counterexample to the significant THg and length relationship seen in the overall analysis. They are an exotic, invasive predatory species in the wider Caribbean Region introduced from the Indo-Pacific (Froese and Pauly 2023). However, the mean THg concentration of 0.08 [0.024, 0.50] µg/g ww is relatively low for fish of their size (mean length in this study=28.4 cm; n = 46) and trophic level (4.4; Froese and Pauly 2023). The range of THg concentrations are similar to other studies on lionfish in the region with Huge et al. (2014) reporting a range of 0.03 to 0.48 µg/g ww. Intra- and inter-species variation in THg concentrations can be related to a suite of factors (e.g., trophic level, physiology, food quality or availability) (Vander Zanden and Rasmussen 1996; Harris and Bodaly 1998; Trudel and Rasmussen 2006; Dijkstra et al. 2013; Karimi et al. 2016; Eagles-Smith et al. 2018). One possibility is that the invasive lionfish are relatively fast growing in the region compared to their native range and compared to other species in the wider Caribbean Region (Côté et al. 2013; Pusack et al. 2016). This elevated growth rate may be leading to ‘growth dilution’ effects on THg concentrations, although more research is needed for definite attribution of a mechanism (Harris and Bodaly 1998; Trudel and Rasmussen 2006; Ward et al. 2010; Dang and Wang 2012). Notably, there was not a significant relationship between THg and length in lionfish (*F*_*1,44*_ = 0.010, p > 0.05) unlike the modeled results for all fish in the wider Caribbean Region. Although the sampled lionfish had a relatively wide range of lengths (28.4 [7.5, 51] cm), more samples from the extremes of this distribution would be informative. Effective strategies for managing lionfish populations in the wider Caribbean Region will likely include capture and consumption, so understanding the THg concentrations and the processes that impact variation are important factors to consider in the promotion of these campaigns.

Three of the top four families for mean non-standardized THg concentrations are shark families. However, of these three families, the mean length for Ginglymostomatidae (nurse sharks; geometric mean THg = 0.78 µg/g ww) and Sphyrnidae (bonnethead and hammerhead sharks; 0.76 µg/g ww) were 160 and 106 cm, respectively, the two highest mean length values sampled (no length measurement was available for the lone Alopiidae [thresher sharks]). After accounting for the confounding factors, Ginglymostomatidae and Sphyrnidae had model predicted THg concentrations of 0.19 µg/g ww and 0.25 µg/g ww. This pattern, combined with residuals that indicate the model is a good or slightly overpredicted THg concentrations, is consistent with bioaccumulation and biomagnification with larger, older, higher trophic level individuals sampled. Sharks are large, long-lived meso to apex predators and are reported to accumulate high levels of mercury (Goyanna et al. 2023). Although not wide-spread in all countries, shark consumption does occur in some places, including in the wider Caribbean Region. Shark consumption is particularly high in Trinidad and Tobago, which harvests 488 tonnes annually, much of it for local consumption (Mohammed and Mohammed 2017). The elevated THg concentrations reported here indicate a potential risk to human health from continual consumption of sharks and highlights the importance of informed decision-making in terms of the consumption frequency for some individuals. Additionally, elevated THg concentrations can have adverse impacts on fitness and reproductive success in many species, which impacts population-level dynamics. The elevated mercury concentrations reported here are consistent with levels documented to cause sub-lethal adverse effects, including behavioral (e.g., predator avoidance, gross motor function), physiological (e.g., reduced growth rates, mass) and reproductive (e.g, reduced spawning success and behavior, fecundity) in other fish species (Webber and Haines 2003; Depew et al. 2012; Scheuhammer et al. 2012, 2015; Carvan et al. 2017). As a result, mercury levels may be an additional factor threatening the populations of shark species in addition to those more commonly associated with declining shark populations (e.g., intentional and accidental [by-catch] harvest, climate change). Many shark species, including some of those found in the wider Caribbean Region, are already considered at risk for extinction, so understanding patterns in THg concentrations can inform conservation efforts (Dulvy et al. 2014). The elevated THg levels in some shark species highlights the importance of awareness raising campaigns in the wider Caribbean Region, so that consumers can make informed choices regarding consumption and conservation efforts can account for additional population stressors.

## Conclusion

We found wide variability in mercury concentrations among individuals, species, families, sampling country, fish length, and trophic level in the wider Caribbean Region. Patterns in mercury concentrations are important for understanding toxicological impacts on biota and human health (e.g. neurological impairment, reduced reproductive success, survival). However, these concentrations are driven by a complex suite of factors. We found significant relationships between total mercury concentrations and taxonomic family, sampling country, fish length, and trophic level in fish sampled in the wider Caribbean Region. The data presented here indicate that 55% of individual samples were below 0.23 µg/g ww guideline from the U.S. FDA/EPA (2022) for 2 or 3 weekly servings. However, 26% exceeded the 0.46 µg/g ww guideline as a choice to avoid and consistent with adverse effects on human health from continual consumption, particularly for sensitive populations. This supports a need for further sampling with more concrete geospatial data to better understand patterns and mechanisms in mercury concentrations and allow for more informed decision making on the consumption of fish and invertebrates from the wider Caribbean Region.

### Supplementary information

Supplementary Information

## References

[CR1] Ackerman JT, Eagles-Smith CA, Herzog MP (2016). Avian mercury exposure and toxicological risk across western North America: A synthesis. Sci Total Environ.

[CR2] Adams DH, McMichael Jr RH, Henderson GE (2003) Mercury levels in marine and estuarine fishes of Florida 1989–2001. Florida Marine Research Institute Technical Report TR-9. 2nd ed. rev. 57

[CR3] AMAP/UN Environment (2019). Technical Background Report for the Global Mercury Assessment 2018.

[CR4] Amos HM, Jacob DJ, Streets DG, Sunderland EM (2013). Legacy impacts of all‐time anthropogenic emissions on the global mercury cycle. Global Biogeochemical Cycles.

[CR5] Asner GP, Llactayo W, Tupayachi R, Luna ER (2013). Elevated rates of gold mining in the Amazon revealed through high-resolution monitoring. Proc Natl Acad Sci.

[CR6] Balogh SJ, Meyer ML, Johnson DK (1998). Transport of mercury in three contrasting river basins. Environ Sci Technol.

[CR7] Bastos WR, Dórea JG, Bernardi JVE, Lauthartte LC, Mussy MH, Lacerda LD, Malm O (2015). Mercury in fish of the Madeira river (temporal and spatial assessment), Brazilian Amazon. Environ Res.

[CR8] Basu N, Horvat M, Evers DC, Zastenskaya I, Weihe P, Tempowski J (2018). A state-of-the-science review of mercury biomarkers in human populations worldwide between 2000 and 2018. Environ Health Perspect.

[CR9] Bates D, Maechler B, Bolker, Walker S (2015). Fitting linear mixed effects models using lme4. J Stat Softw.

[CR10] BCRC-Caribbean (2021). Antigua and Barbuda Minamata Initial Assessment Report.

[CR11] Beltrán Turriago C, Mateo J, Blanc PP, del Rio Poza A (2023). The mahi-mahi value chain in the Dominican Republic: Summary Report.

[CR12] Bloom NS (1992). On the chemical form of mercury in edible fish and marine invertebrate tissue. Can J Fish Aquat Sci.

[CR13] Braaten HFV, Fjeld E, Rognerud S, Lund E, Larssen T (2014). Seasonal and year‐to‐year variation of mercury concentration in perch (Perca fluviatilis) in boreal lakes. Environ Toxicol Chem.

[CR14] Braaten HFV, de Wit HA, Larssen T, Poste AE (2018). Mercury in fish from Norwegian lakes: the complex influence of aqueous organic carbon. Sci Total Environ.

[CR15] Braaten HFV, Åkerblom S, Kahilainen KK, Rask M, Vuorenmaa J, Mannio J, Malinen T, Lydersen E, Poste AE, Amundsen PA, Kashulin N (2019). Improved environmental status: 50 years of declining fish mercury levels in boreal and subarctic Fennoscandia. Environ Sci Technol.

[CR120] Bodaly RA, Jansen WA, Majewski AR, Fudge RJ, Strange NE, Derksen AJ, Green DJ (2007) Postimpoundment time course of increased mercury concentrations in fish in hydroelectric reservoirs of northern Manitoba, Canada. Arch Environ Contamination Toxicol 53:379–389 10.1007/s00244-006-0113-4 17728990

[CR17] Boettiger C, Lang DT, Wainwright PC (2012). rfishbase: exploring, manipulating and visualizing FishBase data from R. J Fish Biol.

[CR18] Caballero Espejo J, Messinger M, Román-Dañobeytia F, Ascorra C, Fernandez LE, Silman M (2018). Deforestation and forest degradation due to gold mining in the Peruvian Amazon: A 34-year perspective. Remote Sensing.

[CR19] Cartagena Convention on the Protection and Development of the Marine Environment of the Wider Caribbean Region (1983)

[CR20] Carvan MJ, Kalluvila TA, Klingler RH, Larson JK, Pickens M, Mora-Zamorano FX, Connaughton VP, Sadler-Riggleman I, Beck D, Skinner MK (2017). Mercury-induced epigenetic transgenerational inheritance of abnormal neurobehavior is correlated with sperm epimutations in zebrafish. PLoS ONE.

[CR21] Chen CY, Dionne M, Mayes BM, Ward DM, Sturup S, Jackson BP (2009). Mercury Bioavailability and Bioaccumulation in Estuarine Food Webs in the Gulf of Maine. Env Sci Technol.

[CR22] Chen CY, Buckman KL, Shaw A, Curtis A, Taylor M, Montesdeoca M, Driscoll C (2021). The influence of nutrient loading on methylmercury availability in Long Island estuaries. Environ Pollut.

[CR23] Côté IM, Green SJ, Hixon MA (2013). Predatory fish invaders: insights from Indo-Pacific lionfish in the western Atlantic and Caribbean. Biol Conserv.

[CR24] Crespo-Lopez ME, Augusto-Oliveira M, Lopes-Araújo A, Santos-Sacramento L, Takeda PY, de Matos Macchi B, do Nascimento JL, Maia CS, Lima RR, Arrifano GP (2021). Mercury: What can we learn from the Amazon?. Environ Int.

[CR25] CRFM (2021). CRFM Statistics and Information Report – 2020.

[CR26] Dang F, Wang W-X (2012). Why mercury concentration increases with fish size? Biokinetic explanation. Environ Pollut.

[CR28] Depew DC, Basu N, Burgess NM, Campbell LM, Devlin EW, Drevnick PE, Hammerschmidt CR, Murphy CA, Sandheinrich MB, Wiener JG (2012). Toxicity of dietary methylmercury to fish: derivation of ecologically meaningful threshold concentrations. Environ Toxicol Chem.

[CR29] Dijkstra JA, Buckman KL, Ward D, Evans DW, Dionne M, Chen CY (2013). Experimental and natural warming elevates mercury concentrations in estuarine fish. PloS One.

[CR30] Diringer SE, Berky AJ, Marani M, Ortiz EJ, Karatum O, Plata DL, Pan WK, Hsu-Kim H (2020). Deforestation due to artisanal and small-scale gold mining exacerbates soil and mercury mobilization in Madre de Dios, Peru. Environ Sci Technol.

[CR31] Driscoll CT, Chen CY, Hammerschmidt CR, Mason RP, Gilmour CC, Sunderland EM, Greenfield BK, Buckman KL, Lamborg CH (2012). Nutrient supply and mercury dynamics in marine ecosystems: A conceptual model. Environ Res.

[CR32] Driscoll CT, Mason RP, Chan HM, Jacob DJ, Pirrone N (2013). Mercury as a global pollutant: sources, pathways, and effects. Environ Sci Technol.

[CR33] Dulvy NK, Fowler SL, Musick JA, Cavanagh RD, Kyne PM, Harrison LR, Carlson JK, Davidson LN, Fordham SV, Francis MP, Pollock CM (2014). Extinction risk and conservation of the world’s sharks and rays. elife.

[CR34] Eagles‐Smith CA, Ackerman JT, Adelsbach TL, Takekawa JY, Miles AK, Keister RA (2008). Mercury correlations among six tissues for four waterbird species breeding in San Francisco Bay, California, USA. Environ Toxicol Chem.

[CR35] Eagles-Smith CA, Ackerman JT (2009). Rapid changes in small fish mercury concentrations in estuarine wetlands: implications for wildlife risk and monitoring programs. Environ Sci Technol.

[CR36] Eagles-Smith CA, Wiener JG, Eckley CS, Willacker JJ, Evers DC, Marvin-DiPasquale M, Obrist D, Fleck JA, Aiken GR, Lepak JM, Jackson AK (2016). Mercury in western North America: A synthesis of environmental contamination, fluxes, bioaccumulation, and risk to fish and wildlife. Sci Total Environ.

[CR37] Eagles-Smith CA, Silbergeld EK, Basu N, Bustamante P, Diaz-Barriga F, Hopkins WA, Kidd KA, Nyland JF (2018). Modulators of mercury risk to wildlife and humans in the context of rapid global change. Ambio.

[CR38] Evers DC (2018). The effects of methylmercury on wildlife: a comprehensive review and approach for interpretation. Encyclopedia Anthropocene.

[CR39] FAO/WHO Expert Committee on Food Additives. Meeting (67th: 2006: Rome, Italy), World Health Organization & Food and Agriculture Organization of the United Nations. (2007). Evaluation of certain food additives and contaminants: sixty-seventh report of the Joint FAO/WHO Expert Committee on Food Additives. World Health Organization

[CR40] FAO (2010) The State of World Fisheries and aquaculture 2010. Rome

[CR41] FAO (2020). The State of World Fisheries and Aquaculture 2020.

[CR42] FAO (2022). The State of World Fisheries and Aquaculture 2022. Towards Blue Transformation.

[CR43] Froese R, Pauly D, Editors (2023) FishBase. World Wide Web electronic publication. www.fishbase.org, version (02/2023)

[CR44] Gerson JR, Szponar N, Zambrano AA, Bergquist B, Broadbent E, Driscoll CT, Erkenswick G, Evers DC, Fernandez LE, Hsu-Kim H, Inga G (2022). Amazon forests capture high levels of atmospheric mercury pollution from artisanal gold mining. Nat Commun.

[CR45] Gilmour CC, Podar M, Bullock AL (2013). Mercury methylation by novel microorganisms from new environments. Environ Sci Technol.

[CR46] Girvan AST (2021). Examination of public policy and private sector purchasing practices to improve consumption and intra-region trade of seafood for the Caribbean small-scale fisheries sector.

[CR47] Government of Suriname (2020) Suriname Minamata Initial Assessment Report 2020

[CR27] Goyanna FAA, Fernandes MB, de Silva GB, de Lacerda LD (2023). Mercury in oceanic upper trophic level sharks and bony fishes-A systematic review.. Environ Pollut.

[CR48] Grieb TM, Driscoll C, Gloss S, Schofield C, Bowie G, Porcella D (1990). Factors affecting mercury accumulation in fish in the Upper Michigan Peninsula. Environ Toxicol Chem.

[CR49] Grieb TM, Fisher NS, Karimi R, Levin L (2020). An assessment of temporal trends in mercury concentrations in fish. Ecotoxicology.

[CR50] Gustin M, Evers DC, Bank MS, Hammerschmidt CR, Pierce A, Basu N, Blum J, Bustamante P, Chen C, Driscoll CT, Horvat M (2016). Importance of integration and implementation of emerging and future mercury research into the Minamata Convention. Environ Sci Technol.

[CR51] Hall BD, Bodaly RA, Fudge RJP, Rudd JWM, Rosenberg DM (1997). Food as the dominant pathway of methylmercury uptake by fish. Water Air Soil Pollut.

[CR52] Hammerschmidt CR, Fitzgerald WF (2006). Bioaccumulation and trophic transfer of methylmercury in Long Island Sound. Arch Environ Contamination Toxicol.

[CR53] Harris RC, Bodaly RA (1998). Temperature, growth and dietary effects on fish mercury dynamics in two Ontario lakes. Biogeochemistry.

[CR54] Hsu-Kim H, Kucharzyk KH, Zhang T, Deshusses MA (2013). Mechanisms regulating mercury bioavailability for methylating microorganisms in the aquatic environment: a critical review. Environ Sci Technol.

[CR55] Hsu-Kim H, Eckley CS, Achá D, Feng X, Gilmour CC, Jonsson S, Mitchell CP (2018). Challenges and opportunities for managing aquatic mercury pollution in altered landscapes. Ambio.

[CR56] Huge DH, Schofield PJ, Jacoby CA, Frazer TK (2014). Total mercury concentrations in lionfish (Pterois volitans/miles) from the Florida Keys National Marine Sanctuary, USA. Marine Pollut Bull.

[CR57] Integrated Taxonomic Information System (ITIS) (2023) www.itis.gov, CC0. 10.5066/F7KH0KBK

[CR58] Karagas MR, Choi AL, Oken E, Horvat M, Schoeny R, Kamai E, Cowell W, Grandjean P, Korrick S (2012). Evidence on the human health effects of low-level methylmercury exposure. Environ Health Perspect.

[CR59] Karimi R, Chen CY, Folt CL (2016). Comparing nearshore benthic and pelagic prey as mercury sources to lake fish: the importance of prey quality and mercury content. Sci Total Environ.

[CR60] Kocman D, Horvat M, Pirrone N, Cinnirella S (2013). Contribution of contaminated sites to the global mercury budget. Environ Res.

[CR61] Kocman D, Wilson SJ, Amos HM, Telmer KH, Steenhuisen F, Sunderland EM, Mason RP, Outridge P, Horvat M (2017). Toward an assessment of the global inventory of present-day mercury releases to freshwater environments. Int J Environ Res Public Health.

[CR62] Koenig CC, Coleman FC, Eklund AM, Schull J, Ueland J (2007). Mangroves as essential nursery habitat for goliath grouper (*Epinephelus itajara*). Bull Marine Sci.

[CR63] Lenth R (2023) emmeans: Estimated Marginal Means, aka Least-Squares Means. R package version 1.8.4-1, https://CRAN.R-project.org/package=emmeans

[CR64] Lavoie RA, Jardine TD, Chumchal MM, Kidd KA, Campbell LM (2013). Biomagnification of mercury in aquatic food webs: a worldwide meta-analysis. Environ Sci Technol.

[CR65] Lescord GL, Johnston TA, Branfireun BA, Gunn JM (2018). Percentage of methylmercury in the muscle tissue of freshwater fish varies with body size and age and among species. Environ Toxicol Chem.

[CR66] Liu M, Zhang Q, Maavara T, Liu S, Wang X, Raymond PA (2021). Rivers as the largest source of mercury to coastal oceans worldwide. Nat Geosci.

[CR67] Maldonado JH, Sánchez RDPM, Morales MEV, Leguízamo E (2022). Livelihoods characterization of a small-scale fishing community in the Colombian caribbean. Marine Fishery Sci.

[CR68] Malinowski CR (2019). High mercury concentrations in Atlantic Goliath Grouper: spatial analysis of a vulnerable species. Marine Poll Bull.

[CR69] Mason RP, Abbott ML, Bodaly RA, Bullock OR, Driscoll CT, Evers DC, Lindberg SE, Murray M, Swain EB (2005). Monitoring the response to changing mercury deposition. Environ Sci Technol.

[CR70] Mohammed A, Mohammed T (2017). Mercury, arsenic, cadmium and lead in two commercial shark species (Sphyrna lewini and Caraharinus porosus) in Trinidad and Tobago. Marine Pollut Bull.

[CR71] Obrist D, Kirk JL, Zhang L, Sunderland EM, Jiskra M, Selin NE (2018). A review of global environmental mercury processes in response to human and natural perturbations: Changes of emissions, climate, and land use. Ambio.

[CR72] Outridge PM, Mason RP, Wang F, Guerrero S, Heimburger-Boavida LE (2018). Updated global and oceanic mercury budgets for the United Nations Global Mercury Assessment 2018. Environ Sci Technol.

[CR73] Pereira G, Josupeit H (2017) The world lobster market. In Globefish Research Programme; Food and Agricultural Organization: Rome, Italy, 2017; Volume 123, 41p

[CR74] Petre SJ, Sackett DK, Aday DD (2012). Do national advisories serve local consumers: an assessment of mercury in economically important North Carolina fish. J Environ Monit.

[CR75] Piraino MN, Taylor DL (2009). Bioaccumulation and trophic transfer of mercury in striped bass (Morone saxatilis) and tautog (Tautoga onitis) from the Narragansett Bay (Rhode Island, USA). Marine Environ Res.

[CR76] Podar M, Gilmour CC, Brandt CC, Soren A, Brown SD, Crable BR, Palumbo AV, Somenahally AC, Elias DA (2015). Global prevalence and distribution of genes and microorganisms involved in mercury methylation. Sci Adv.

[CR77] Pusack TJ, Benkwitt CE, Cure K, Kindinger TL (2016). Invasive Red Lionfish (Pterois volitans) grow faster in the Atlantic Ocean than in their native Pacific range. Environ Biol Fishes.

[CR78] R Core Team (2023) R: A language and environment for statistical computing. R Foundation for Statistical Computing, Vienna, Austria, https://www.R-project.org/

[CR79] Rice KM, Walker EM, Wu M, Gillette C, Blough ER (2014). Environmental mercury and its toxic effects. J Prevent Med Public Health.

[CR80] Richter W, Skinner LC (2020). Mercury in the fish of New Yorkʼs Great Lakes: A quarter century of near stability. Ecotoxicology.

[CR81] Ricketts P, Basu N, Fletcher H, Voutchkov M, Bassaw B (2016). Assessment of fish consumption and mercury exposure among pregnant women in Jamaica and Trinidad & Tobago. Chemosphere.

[CR82] Rojas de Astudillo L, Chang Yen I, Bekele I (2005). Heavy metals in sediments, mussels and oysters from Trinidad and Venezuela. Revista de biología tropical.

[CR83] Rolfhus KR, Hall BD, Monson BA, Paterson MJ, Jeremiason JD (2011). Assessment of mercury bioaccumulation within the pelagic food web of lakes in the western Great Lakes region. Ecotoxicology.

[CR84] Roué-Legall A, Lucotte M, Carreau J, Canuel R, Garcia E (2005). Development of an ecosystem sensitivity model regarding mercury levels in fish using a preference modeling methodology: Application to the Canadian boreal system. Environ Sci Technol.

[CR85] Roulet M, Lucotte M, Canuel R, Farella N, Courcelles M, Guimaraes JR, Mergler D, Amorim M (2000). Increase in mercury contamination recorded in lacustrine sediments following deforestation in the central Amazon. Chem Geology.

[CR86] Sackett DK, Cope WG, Rice JA, Aday DD (2013). The influence of fish length on tissue mercury dynamics: implications for natural resource management and human health risk. Int J Environ Res Public Health.

[CR87] Scheuhammer AM, Basu N, Evers DC, Heinz G, Sandheinrich MB, Bank MS, Bank M (2012). Ecotoxicology of mercury in fish and wildlife: Recent advances. Mercury in the Environment: Pattern and Process.

[CR88] Scheuhammer A, Braune B, Chan HM, Frouin H, Krey A, Letcher R, Loseto L, Noël M, Ostertag S, Ross P, Wayland M (2015). Recent progress on our understanding of the biological effects of mercury in fish and wildlife in the Canadian Arctic. Sci Total Environ.

[CR89] Scudder BC, Chasar LC, DeWeese LR, Brigham ME, Wentz DA, Brumbaugh WG (2008) Procedures for collecting and processing aquatic invertebrates and fish for analysis of mercury as part of the National Water-Quality Assessment Program: U.S. Geological Survey Open-File Report 2008–1208, 34 p

[CR90] Shanley JB, Moore R, Smith RA, Miller EK, Simcox A, Kamman N, Nacci D, Robinson K, Johnston JM, Hughes MM, Johnston C (2012). MERGANSER: an empirical model to predict fish and loon mercury in New England lakes. Environ Sci Technol.

[CR91] Simoneau M, Lucotte M, Garceau S, Laliberté D (2005). Fish growth rates modulate mercury concentrations in walleye (Sander vitreus) from eastern Canadian lakes. Environ Res.

[CR92] Somers KM, Jackson DA (1993). Adjusting mercury concentration for fish-size covariation: a multivariate alternative to bivariate regression. Can J Fish Aquatic Sci.

[CR93] Spanier E, Lavalli KL, Goldstein JS, Groeneveld JC, Jordaan GL, Jones CM, Phillips BF, Bianchini ML, Kibler RD, Díaz D, Mallol S (2015). A concise review of lobster utilization by worldwide human populations from prehistory to the modern era. ICES J Marine Sci.

[CR94] Stephen AL, Murray P (2008) International trade and fisheries: Implications for fisheries management and development of small vulnerable Caribbean states. Proceedings of the 61st Gulf and Caribbean Fisheries Institute 61

[CR95] Streets DG, Horowitz HM, Jacob DJ, Lu Z, Levin L, Ter Schure AF, Sunderland EM (2017). Total mercury released to the environment by human activities. Environ Sci Technol.

[CR96] Sunderland EM (2007). Mercury exposure from domestic and imported estuarine and marine fish in the U.S. seafood market. Environ Health Perspect.

[CR97] Sunderland EM, Li M, Bullard K (2018). Decadal changes in the edible supply of seafood and methylmercury exposure in the United States. Environ Health Perspect.

[CR98] Swenson JJ, Carter CE, Domec JC, Delgado CI (2011). Gold mining in the Peruvian Amazon: global prices, deforestation, and mercury imports. PloS One 2011.

[CR99] Taylor VF, Buckman KL, Seelen EA, Mazrui NM, Balcom PH, Mason RP, Chen CY (2019). Organic carbon content drives methylmercury levels in the water column and in estuarine food webs across latitudes in the Northeast United States. Environ Pollut.

[CR100] Teh LC, Ota Y, Cisneros‐Montemayor AM, Harrington L, Swartz W (2020). Are fishers poor? Getting to the bottom of marine fisheries income statistics. Fish Fisheries.

[CR101] Tewfik A, Babcock EA, Phillips M (2020). Spiny lobster fisheries status across time and a mosaic of spatial management regimes. ICES J Marine Sci.

[CR102] Travnikov O, Angot H, Artaxo P, Bencardino M, Bieser J, d’Amore F, Dastoor A, De Simone F, Diéguez MDC, Dommergue A, Ebinghaus R (2017). Multi-model study of mercury dispersion in the atmosphere: atmospheric processes and model evaluation. Atmos Chem Phys.

[CR103] Trudel M, Rasmussen JB (2006). Bioenergetics and mercury dynamics in fish: a modelling perspective. Can J Fish Aquatic Sci.

[CR104] United Nations Environment Programme (UNEP) (2013) Minamata convention on mercury: Texts and annexes. UNEP Chemicals Branch, Geneva, Switzerland

[CR105] United Nations Environment Programme (UNEP) (2019) Global Mercury Assessment 2018. UN Environment Programme. Chemicals and Health Branch, Geneva, Switzerland

[CR107] U.S. EPA (1998) Method 7473 (SW-846): Mercury in Solids and Solutions by Thermal Decomposition, Amalgamation, and Atomic Absorption Spectrophotometry. Revision 0. Washington, DC.

[CR108] U.S. EPA (2000). Guidance for assessing chemical contaminant data for use in fish advisories. Fish Sampling and Analysis, 3rd edition volume 1.

[CR106] U.S. FDA / EPA (2022) Technical Information on Development of FDA/EPA Advice about Eating Fish for Those Who Might Become or Are Pregnant or Breastfeeding and Children Ages 1-11 Years. https://www.fda.gov/food/metals-and-your-food/technical-information-development-fdaepa-advice-about-eating-fish-those-who-might-become-or-are

[CR109] Van Walleghem JL, Blanchfield PJ, Hintelmann H (2007). Elimination of mercury by yellow perch in the wild. Environ Sci Technol.

[CR110] Vander Zanden MJ, Rasmussen JB (1996). A trophic position model of pelagic food webs: impact on contaminant bioaccumulation in lake trout. Ecol Monogr.

[CR111] Walters DM, Blocksom KA, Lazorchak JM, Jicha T, Angradi TR, Bolgrien DW (2010). Mercury contamination in fish in midcontinent great rivers of the United States: Importance of species traits and environmental factors. Environ Sci Technol.

[CR112] Ward DM, Nislow KH, Chen CY, Folt CL (2010). Rapid, efficient growth reduces mercury concentrations in stream-dwelling Atlantic salmon. Trans Am Fish Soc.

[CR113] Webber HM, Haines TA (2003). Mercury effects on predator avoidance behavior of a forage fish golden shiner (Notemigonus crysoleucas). Environ Toxicol Chem.

[CR114] Weiner J, Krabbenhoft D, Heinz G, Scheuhammer AM, Hoffman DJ, Rattner BA, Burton GAJ, Cairns JJ (2003). Ecotoxicology of Mercury. Handbook of Ecotoxicology.

[CR115] Winemiller KO, Rose KA (1991). Patterns of life-history diversification in North American fishes: implications for population regulation. Can J Fish Aquatic Sci.

[CR116] WHO (2020) 10 chemicals of public health concern

[CR117] Wu P, Kainz MJ, Bravo AG, Åkerblom S, Sonesten L, Bishop K (2019). The importance of bioconcentration into the pelagic food web base for methylmercury biomagnification: A meta-analysis. Sci Total Environ.

[CR118] Wyn B, Kidd KA, Burgess NM, Curry RA, Munkittrick KR (2010). Increasing mercury in yellow perch at a hotspot in Atlantic Canada, Kejimkujik National Park. Environ Sci Technol.

[CR119] Yoshimura A, Suemasu K, Veiga MM (2021). Estimation of mercury losses and gold production by artisanal and small-scale gold mining (ASGM). J Sustain Metallurgy.

