# Supplementary material for: An evaluation of fish and invertebrate mercury concentrations in the Caribbean Region
Source: Ecotoxicology. 2024 Jun 5;33(4-5):397–414. doi: 10.1007/s10646-024-02754-y (PMC11213769; doi:10.1007/s10646-024-02754-y)
Supplement: Supplementary file 1 — Supplementary Information [file 10646_2024_2754_MOESM1_ESM.docx]

**Supplementary Information**

**An evaluation of fish and invertebrate mercury concentrations in the Caribbean Region**

*Ecotoxicology*

**Authors**: Linroy Christian^1*^, Mark E.H. Burton, Azad Mohammed, Wendy Nelson, Tahlia Ali Shah, Laël Bertide-Josiah, Helen G. Yurek, David C. Evers

**Corresponding Autho**r: Linroy Christian^1*^

^1^Ministry of Foreign Affairs, Agriculture, Trade and Barbuda Affairs, St. John's, Antigua and Barbuda

*Linroy.Christian@ab.gov.ag

**Fig. S1** Residuals vs predicted fish total mercury (THg) plot for the mixed effects model

**Fig. S2** Actual vs predicted fish total mercury (THg) plot for the mixed effects model


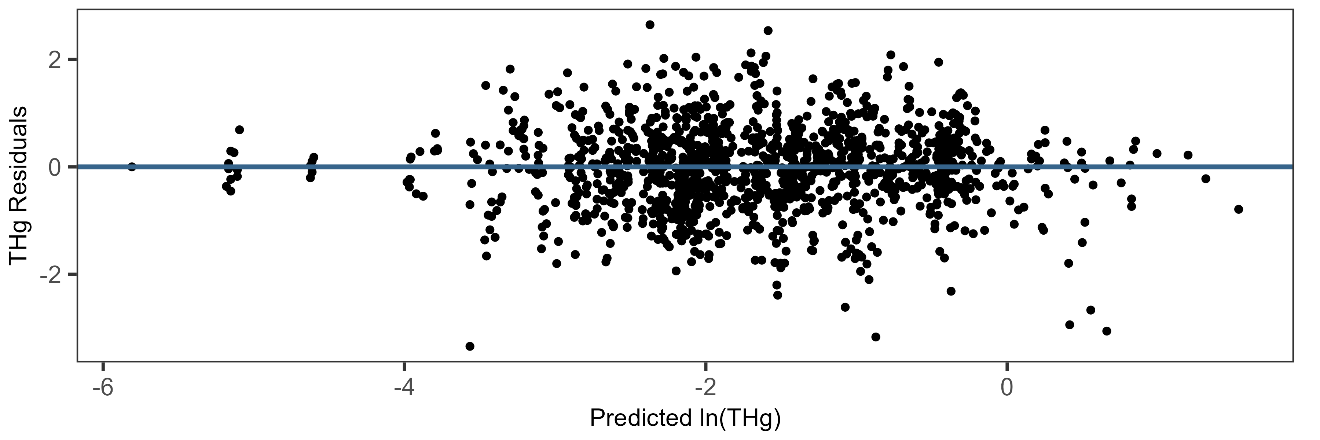


**Fig. S1** Residuals vs predicted fish total mercury (THg) plot for the mixed effects model


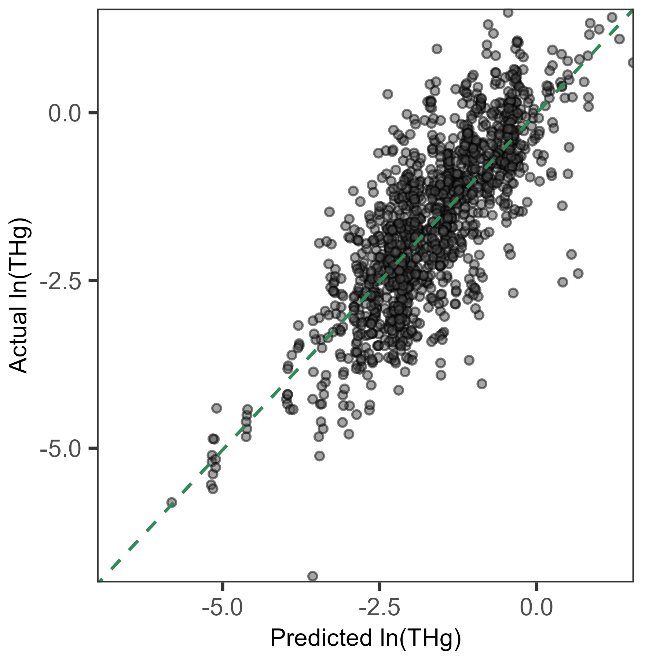


**Fig. S2** Actual vs predicted fish total mercury (THg) plot for the mixed effects model
